# Primary care provider notions on instituting community-based geriatric support in Uganda

**DOI:** 10.1186/s12877-022-02897-9

**Published:** 2022-03-29

**Authors:** Jude Thaddeus Ssensamba, Mary Nakafeero, Hellen Musana, Mathew Amollo, Aloysius Ssennyonjo, Suzanne N. Kiwanuka

**Affiliations:** 1 Division of Infectious Diseases and Geriatric Health, Center for Innovations in Health Africa, Kampala, Uganda; 2 Health Care Programmes, VIVES University of Applied Sciences, Kortrijk, Belgium; 3 School of Public Health, Makerere University College of Health Sciences, Kampala, Uganda; 4 School of Public Health, Department of Epidemiology and Biostatistics, Makerere University College of Health Sciences, Kampala, Uganda; 5 School of Public Health, Department of Health Policy and Planning, Makerere University College of Health Sciences, Kampala, Uganda

**Keywords:** Community-based geriatric support, Uganda, Health providers, Notions, Old adults, Primary health care, The elderly

## Abstract

**Background:**

Understanding of the most economical and sustainable models of providing geriatric care to Africa’s rising ageing population is critical. In Uganda, the number of old adults (60 years and above) continues to rise against absence of policies and guidelines, and models for providing care to this critical population. Our study explored public primary health care provider views on how best community-based geriatric support (CBGS) could be instituted as an adaptable model for delivering geriatric care in Uganda’s resource-limited primary public health care settings.

**Methods:**

We interviewed 20 key informants from four districts of Bukomansimbi, Kalungu, Rakai, and Lwengo in Southern Central Uganda. Respondents were leads (in-charges) of public primary health units that had spent at least 6 months at the fore said facilities. All interviews were audio-recorded, transcribed verbatim, and analysed based on Hsieh and Shannon’s approach to conventional manifest content analysis.

**Results:**

During analysis, four themes emerged: 1) Structures to leverage for CBGS, 2) How to promote CBGS, 3) Who should be involved in CBGS, and 4) What activities need to be leveraged to advance CBGS? The majority of the respondents viewed using the existing village health team and local leadership structures as key to the successful institutionalization of CBGS; leveraging community education and sensitization using radio, television, and engaging health workers, family relatives, and neighbors. Health outreach activities were mentioned as one of the avenues that could be leveraged to provide CBGS.

**Conclusion:**

Provider notions pointed to CBGS as a viable model for instituting geriatric care in Uganda’s public primary healthcare system. However, this requires policymakers to leverage existing village health team and local governance structures, conduct community education and sensitization about CBGS, and bring onboard health workers, family relatives, and neighbors.

**Supplementary Information:**

The online version contains supplementary material available at 10.1186/s12877-022-02897-9.

## Background

The world will be home to 2.1 billion old adults by 2050 [1]. Africa’s ageing population is expected to be home to 10% of this population [2]. In Uganda, the population of the elderly (people aged 60 years and above) is expected to be 5.8% of its total population by then [2]. Ageing comes with unique economic [3, 4] physical, psychological, and health vulnerabilities [5–8]. This, in line with the Universal Health Coverage’s “leave no one behind” agenda calls for government efforts to design quality, accessible, and equitable promotive, preventive, curative, rehabilitative and palliative health services [9] that effectively and efficiently meet the needs of old adults, at every level of development, if we are to achieve sustainable development goal three. For this, health systems in the developing world should be investing and researching on how best to institute geriatric care into their health system.

Like majority of other developing countries, Uganda has no policy, guidelines, or models to guide geriatric practice. Existing efforts are mainly private and not regulated [10], leaving a gap on how best geriatric care can be introduced into Uganda’s primary health care facilities. In developed countries where the geriatric practice is more advanced, geriatric care draws from mainly two schools; residential care, where old adults are taken to reception centers (geriatric homes) to receive holistic care services [11, 12], and community based geriatric support/care (CBGS), where old persons stay within communities with family members and neighbours. In the latter case, community-based health personnel play a supportive role in their care provision [13–15]. CBGS leverages a patient-centred care approach and enables family and community members to actively take part in caring for their loved ones [13]. Much as this model requires some level of training for family members, it is credited for significantly reducing the costs of care [16, 17], making it a suitable choice for resource limited countries like Uganda. Furthermore, the majority of Uganda’s old adults stay in the rural setting, making CBGS a more practical, socially, and culturally acceptable model [18].

In Uganda, health workers (more so health facility leaders) have been critical in providing ideas and views on how best to start and improve health programs. For example, the success in HIV/AIDs, Tuberculosis, and maternal child health services has been largely based on ideas and strategies from community-based health leadership [19–21]. This is because health workers better understand their community health needs and gaps, are aware of cultural issues surrounding health needs, and are better experienced about how best new programs can be introduced [22, 23]. The purpose of this study was to ascertain primary health care provider notions about instituting community-based geriatric support in Uganda.

## Methods

### Study setting

The study was conducted as part of the CIHA Uganda geriatric health project across public primary health care facilities located in four purposively selected districts of Bukomansimbi, Kalungu, Rakai and Lwengo, which according to the 2014 National census data were home to the highest proportion of older adults in Southern Central Uganda. Presence of a high proportion of older adults in the selected districts was considered vital for understanding how health systems that assumedly interact more with these people were prepared to provide geriatric friendly services, while public health care facilities were selected because they provide health care to the majority of the rural population in Uganda. Details about the study setting, districts, and health facility selection criteria and rationale have been published elsewhere [10].

### Study design

We employed a descriptive qualitative approach [24, 25] to explore views of key informant primary health care providers on how best to institute community-based geriatric support in Uganda. The key informants were purposively selected based on (1) being a health facility in-charge or out-patient department (OPD) in-charge or their delegate; (2) having been at the health facility for at least 6 months; (3) and willingness to be interviewed. By selecting this group of people, we had informants with knowledge and capacity to discuss in detail issues related to health care and the elderly in their catchment area. Newly appointed in-charges (that had spent less than 6 months) were excluded from the study.

### Data collection

The interviews were conducted between February and March of 2018, at public primary health facilities where respondents worked. The interviews were conducted in English, using a piloted semi-structured Key Informant (KI) interview guide. The guide enabled interviewers to probe for clarity and to follow leads that were brought up by the KIs. After obtaining consent from respondents, trained Research Assistants administered the interviews, which lasted an average of 30 min. The respondents were asked about their cadre, length of stay at the health facility, location of health facility, responsibility held at the facility, and open-ended questions on their views about community-based geriatric support. We focused on: structures, promotion, engagement, and activities to leverage for CBGS. Probing questions like “Could you please elaborate more about that?” were used during the interview to elicit detailed responses to the questions at hand. All interviews were audio-recorded and later transcribed verbatim and cross transcribed.

### Data analysis

Data analysis was guided by Hsieh and Shannon’s approach to conventional manifest content analysis [26]. The approach enables one to come up with codes during analysis, is often employed in the health care settings, is well described, and allows one to explore phenomenon through someone else’s eyes in contexts of limited existing scientific literature and knowledge [24, 26, 27]. To explore provider notions about CBGS in Uganda, the researchers first read all transcripts to obtain an overall impression of views presented by participants. The transcripts were then re-read to identify sections of text that were related to CBGS to constitute free codes. After re-reading five transcripts, the researchers developed a preliminary codebook which was revised to include any emerging codes. After enciphering, some codes were merged because of similarity in content, and four major categories emerged. The content from each code group was abstracted and interpretation of coded information was done based on how it relates with other code categories and sub-categories. We achieved convenient saturation as per key informant interview guidelines [28]. The findings derived from analysis are presented in a descriptive format containing summary of findings, and participants’ voice excerpts as quotations.

### Ethical consideration

The study was approved by the Makerere University School of Public Health Higher Degrees Research Ethics Committee (FWA00011353). Before the start of interviews, all key informants (KIs) were taken through the consenting process which included providing verbal and written information pertaining objectives of the study. The consent form also clarified the confidential and anonymous nature of the interview and that participation was voluntary. Key informants had the discretion to withdraw their participation at any time without any consequences. All the 20 respondents gave their verbal and written consent to participate in the study.

## Results

One interview was conducted at each of the 11 HCIIIs, two interviews (with the in-charge and OPD in-charge) at each of the seven HCIVs and hospitals (Table 1). The response rate was 83%, with 20 of 24 anticipated key informants consenting for the interviews. Four interviews could not be conducted because in-charges were not at their health facilities during the data collection period. Table 1 shows that the majority of the key informants were health facility in-charges (*n* = 16, 80%), Medical Clinical Officers by cadre (*n* = 9, 45%), and resided in rural settings (*n* = 10, 50%). There was equal sex distribution among interviewees, although more female health workers (*n* = 7) worked at lower-level health facilities located in rural settings. The average respondents’ age was 33 years (SD ± 7.866). The average time spent at the health facility was 3.8 years, enabling us to interview people with a well-informed community perspective.

**Table 1:** Characteristics of Key informants (*n *= 20)

Variables	Health Facility Level			Total
	HCIII (***n*** = 11)	HCIV (***n*** = 7)	Hospital (***n*** = 2)	***n*** (%)
Cadre of Key informant				
Medical Doctor	0	4	1	5(25)
Medical Clinical officer^a^	9	0	0	9(45)
Nursing Officer	1	2	1	4(20)
Enrolled Nurse	1	1	0	2(10)
Responsibility				
Health facility In-charge	11	4	1	16(80)
OPD In-charge	0	3	1	4(20)
Sex				
Male	4	6	0	10(50)
Female	7	1	2	10(50)
Health facility location				
Urban	1	2	2	5(25)
Semi urban	2	3	0	5(25)
Rural	8	2	0	10 (50)

^a^A rank comparable to Physician Assistants or Assistant Doctors [29]

The four themes that emerged were; Structures to leverage for CBGS; promotion of community-based geriatrics support; who should be involved in community-based geriatric support; and activities to be leveraged to advance the community-based geriatric support agenda. In our narrative, we include the most representative verbalizations.

### Structures for community-based geriatric support

Under this theme, four sub-themes featured prominently, these included; The Village health team (VHT) structure, Local leadership structures, Community-based organizations, and family support structures.

The majority of key informants noted that CBGS would best be implemented through the existing Village Health Teams (VHTs) support structure, a system they reported to have helped them in identifying other groups of interest from the communities for health care and support.

Many respondents also advanced views of training VHTs and local leaders to work together in the provision of community-based geriatric support. Some of the participants had this to say:

*"Currently, we work in partnerships with VHTs. So, I think just like the way they pick out cases of maybe fistula or things like mothers who have [gotten] issues during pregnancy, sexually transmitted diseases (STDs), and all that stuff. I think they can look out for those old people who need a lot of..." (Key Informant, 017)*

*"…Ideally, we have VHTs. The VHTs can work with the local leaders" (Key informant, 003*

Relatedly, a demonstrable number of respondents noted the family structures as another entry point for providing support to old persons, with relatives escorting old persons to health facilities as an example of demonstrating that support.

*"I advise the elderly to come with their relatives when coming to the hospital" (Key informant, 14).*

On the other hand, one respondent fronted the idea of engaging existing community-based organisations, which have already played an instrumental role in supporting existing health structures to improve the quality and content of rural health care.

*"The most reliable method now is through the VHT’s and a few community-based organisations" (Key informant, 005)*

### Promoting community-based geriatric support

Under this theme, two subthemes emerged; creating community awareness and community education.

Participants generally observed that the practice of community-based geriatric support is not well understood and practiced in communities. Even with the existing informal support to older persons at community level, there are gaps in knowledge on likely complications faced by older adults and standards of care to be followed as voiced by one of the participants below:

*"I think this is something to do with giving community education, educating them about the elderly [old people] ...the complications that [come] along with ageing, what we can do, [and] how the community can support them" (Key informant, 007).*

The respondents further noted that creating community awareness and education requires active involvement of health workers and geriatric support organizations to inform and educate locals about this new aspect of care for the elderly within their catchment areas.

*"... the most important thing is that we need to create awareness about geriatric care and how communities can support their old adult from the community…" (Key informant, 005)*

In order to popularize community based geriatric support, leveraging locally available channels for public health promotion and education such as use of local radios were suggested by the participants.

*"...we need to advertise over Television, Radio and use posters to inform communities about the importance of taking care of the elderly, more so in the communities ..." (Key informant, 004).*

### Activities to leverage for the community-based geriatric support

Integrating CBGS activities within existing health outreaches was a popular avenue observed by the participants as key for promoting community-based geriatric support agenda. Through the health outreach activities, the older adults could be reached with tailored health services as well as provide health education to them and their caregivers. Below are some of the participants’ voice excerpts;

*"[Like] some never come to the health facility, they stay in the community [,] but if we have an outreach program, we could look them up and give them the care they need" (Key informant, 015)*

*"... during (our) usual health outreach activities, we can continue highlighting the same [importance of community geriatric support] …" (Key informant, 005),*

Some participants suggested that health workers provide standalone home visits for old adults. This was argued to reduce the inconvenience of older adults trekking long distances to health facilities for health problems that may not require facility based care and treatment.

*"... [Yes] to help those people [the elderly] from where they are [,] so that they don’t go through this bother of going to the health unit every time they get a small problem" (Key informant, 008).*

### Key actors for community-based geriatric support

Much as health workers were noted as the primary players in the provision of community-based geriatric support, family members, and in cases where this is not possible, neighbours were another resource that participants pointed to as key in the provision of geriatric support to old adults. This is illustrated by the following quotations:

*"…I think old people are more comfortable at home than at the health facility, for this engaging their family members and relatives is pertinent..." (Key informant, 006)*

*"...if you are my neighbour, I think we can give attention to you, old people are very important to our society…" (Key informant, 005).*

## Discussion

In this study, we explored key informants’ notions of how to institute community-based geriatric support (CBGS) in Uganda’s primary health care settings. Respondents shared their views on systems structures, promotion, who to engage, and activities for advancing CBGS. Majority of KIs perceived CBGS as the best model for providing care to Uganda’s ageing population. This thinking is based on Uganda’s lack of financial, technical, and logistical capacity to undertake expensive alternatives. We have already noted that Uganda lacks the political will, policy astuteness, leadership capacity, equipment, logistics, and human resource for health, and monitoring and evaluation capacity to institute geriatric care and support in its health care system [10].

The majority of study participants viewed VHTs as the best cadre to implement CBGS. The VHTs are volunteering non-professional community health workers who are located at the lowest level of Uganda’s health system. They are responsible for providing basic health care, sensitization, and health education to people within their locality [30]. The choice of the VHT structure was based on the fact that the respondents have already engaged them successfully to improve maternal-child health services and sexual reproductive services in their catchment areas. The key informant views add to the existing bodies of knowledge that VHTs have been very instrumental in reducing maternal, and childhood mortality and morbidity [31, 32]; improving six-week postnatal follow up of HIV infected women and early infant diagnosis among HIV exposed infants [33]; promoting proper cord and thermal care for new-born babies [34]; improving ART adherence [35], and improving malaria and pneumonia diagnosis and treatment among children [36]. The feasibility of engaging VHTs or community health volunteers as the primary point of contact for CBGS needs to be explored by Uganda, and other developing countries.

Similarly, some key informants suggested engaging and training local leaders to support VHTs in CBGS activities. This notion is construed from their perceived understanding of the health needs of their subjects. Furthermore, local politicians in Uganda have been instrumental in creating awareness, penetrating the community, expediting acceptability and trust of HIV/AIDS, tuberculosis, reproductive, and other communicable and non-communicable disease health programs [37, 38]. Relatedly, the perceived need to engage family members is synonymous with findings from other developing countries like India [1] and the likelihood of social acceptability [18].

Creating community awareness and community education came through as the best avenues for promoting CBGS. This is because community education and awareness give locals within primary health care settings an opportunity to have a deeper understanding of health programs, improves buy-ins, and acceptability of interventions [39]. In Uganda, community awareness creation has been critical in promoting the acceptability of HIV/AIDS care services [40, 41], and improving reporting on medicines-related adverse events [42]. The recommendation to leverage mass media such as television and radio adverts and posters is reminiscent of avenues that Uganda has employed to effectively improve awareness for diseases such as HIV/AIDS, Malaria, and other health services [43–45]. We recommend that policymakers put into consideration the methods proposed by key respondents when promoting CBGS.

Engaging family members and in cases where this is not possible, neighbours were another resource that participants pointed to as key for the provision of support to old adults within their communities. These notions resonate with existing literature that has found engaging family members and the community as critical in improving the quality of life and health outcomes for old adults [46–48]. However, as the government contemplates engaging family members, relatives, and neighbours in CBGS, it is vital that a clear understanding of the financial and social implications of this model to caretakers is given due attention. This is premised on some studies such as one conducted in Ghana that found CBGS to have a demonstrable direct and indirect economic burden to family members [49], though comparatively lower than institutional geriatric care as noted by Xie et al. 2020 [50].

Integrating CBGS within existing health outreach activities was fronted as one of the avenues for advancing the community geriatric support agenda. The integration of health services is less costly, more acceptable since community members receive comprehensive services and adaptable to countries with human resource for health gaps [51–53]. In Uganda, studies have reported health services integration to be vital for improving refugee health services [54], HIV/AIDS and sexual reproductive health among youths in south-western Uganda [55], and mental health [56]. Relatedly, outreach activities have been noted for bringing health services closer to communities, thus reducing health access barriers [57].

### Strengths

Our study was conducted majorly in rural public primary health care facilities where the majority of old adults seek care. For this, opinions expressed by respondents are more likely to be in sync with the aspirations of their communities. Secondly, researchers interviewed a representative sample of respondents, catering for differences in cadre and their experience in providing health services in their catchment areas. This meant that they had a clear understanding of the subject at hand in the context of their communities, making our findings more rigorous. Third, the researchers employed a combination of audio with note-taking, helping us to take note of none verbal aspects from the interviewees and two different people were employed to transcribe same interviews to minimize errors of missing information.

### Limitations

As in all qualitative study designs, we could not draw a statistical conclusion on provider notions or obtain quantifiable data on key variables such as knowledge, attitudes and perceptions among health workers. We therefore recommend quantitative follow-up studies to interrogate this subject.

The researchers also note that our study was limited to southern central Uganda, meaning that provider views may not represent the broader national health care work population. For this, nationwide studies are recommended. Finally, the study was limited to public primary health care facilities, leaving out private health facilities and regional and national referral health care facilities that also provide care to old adults.

## Conclusion and policy implications

The study revealed that instituting CBGS in Uganda’s primary health care system requires the active engagement of VHTs and local leaders since engaging these critical resources will ensure that the program is accepted and well monitored. Relatedly, policy makers must undertake community sensitization and awareness creation through radio, television, and use of other social, behavioural change communication tools such as posters to promote CBGS. Family members, relatives, and neighbours are a vital resource that policymakers need to engage during the provision of CBGS. However, studies should be conducted to estimate the social-economic implications of engaging them and how the effects can be minimised.

## Supplementary Information

**Additional file 1.**

## Data Availability

The datasets generated and/or analysed during the current study are not publicly available due to ethical requirements by the ethics committee. Still, they are available from the corresponding author on reasonable request.

## Abbreviations

CBGS: Community Based Geriatric Support
CIHA: Center for Innovations in Health Africa
HCIII: Health Centre Three
HCIV: Health Centre Four
HF: Health Facility
HFs: Health Facilities
HIV: Human Immunodeficiency Virus
HIV/AIDS: Human Immunodeficiency Virus/Acquired Immunodeficiency Syndrome
HWs: Health workers
KIs: Key Informants
OPD: Outpatient department
SD: Standard Deviation
STDs: Sexually Transmitted Diseases
VHTs: Village Health Teams
