# Supplementary material for: Primary care provider notions on instituting community-based geriatric support in Uganda
Source: BMC Geriatr. 2022 Mar 29;22:258. doi: 10.1186/s12877-022-02897-9 (PMC8962536; doi:10.1186/s12877-022-02897-9)
Supplement: Supplementary file 1 — Additional file 1. [file 12877_2022_2897_MOESM1_ESM.pdf]

Assessing Primary Care Provider Notions on Instituting Community-based Geriatric Support in Uganda

Key informant interview guide

Date of interview: ____________________ Interviewer name: _____________________

Facility name: ____________________ Type/level of facility: ___________________

Location of Health Facility:___________________________ (Rural/Urban/Semi urban)

Introduction

Hello thanks for agreeing to participate in this interview. Just as a reminder, this interview will be exploring your views and experiences about what can be done to institute community-based geriatric support within your community. This interview should take around 30 to 45 minutes of your time but take your time to share all your views as exhaustively as possible. If you have any questions or issues to raise before we start and during the interview, feel free to share with me.

Did the participant raise any questions? □ Yes

Explain briefly

____

____

□ No – Proceed with the interview

Part: 1 I would like to start by asking a few questions about your job.

1. What is your job title? [In-charge, Medical superintendent, Geriatric focal person etc.]

2. What cadre of health worker are you? [Medical Doctor, Medical Clinical officer, Nursing Officer, Enrolled Nurse, etc.]?

3. Respondent's Gender (Male/Female)

4. What is your age? (record these in years)

Part II: Thank you, I will now ask you about your views concerning instituting community-based geriatric care within your community. Community-based geriatric support/care (CBGS) is a state where the elderly stay within communities and receive primary health care within their localities with the support of family members, relatives, and neighbours.

5. As exhaustively as possible, share with me your views about existing structures that can be leveraged to institute, and provide community-based geriatric support to the elderly in your catchment area. (Probe, for more responses until the respondent notes that all views are exhausted).

6. Currently, there is little known about community-based geriatric support in Uganda. Kindly share with me how this practice can be made known in your community. Give examples of what can be done. Be as exhaustive as possible.

7. In your view, who are the key players that should be involved to institute and implement community-based geriatric support? Give as many examples as possible, and explain how and why they are important.

8. Based on prevailing the situation at your health facility, share with me the existing platforms, activities, programs, and frameworks that could be utilized to institute community-based geriatric support in your community. Be as exhaustive as possible.

9. Tell me about any other thing that needs to be given attention with regards to instituting community-based geriatric support in Uganda. Explain your answer please. Be as exhaustive as possible.

Thank you. We have reached the end of the interview. Note that every response given will be treated with confidentiality. You contributions will be critical in helping Uganda institute a sustainable model for providing care to the elderly. If you have additional questions, feel free to ask me now.
